# Stress Levels Escalate When Repeatedly Performing Tasks Involving Threats

**DOI:** 10.3389/fpsyg.2019.01562

**Published:** 2019-07-04

**Authors:** Johan Bertilsson, Diederick C. Niehorster, Peter Jan Fredriksson, Mats Dahl, Simon Granér, Ola Fredriksson, Johan Magnus Mårtensson, Måns Magnusson, Per-Anders Fransson, Marcus Nyström

**Affiliations:** ^1^Police Region South, Swedish Police Authority, Malmö, Sweden; ^2^Department of Clinical Sciences, Lund University, Lund, Sweden; ^3^Lund University Humanities Lab, Lund, Sweden; ^4^Department of Psychology, Lund University, Lund, Sweden; ^5^Lund University Cognitive Science, Lund University, Lund, Sweden

**Keywords:** stress escalation, heart rate, pupil dilation, repetition, operative police tactics

## Abstract

Police work may include performing repeated tasks under the influence of psychological stress, which can affect perceptual, cognitive and motor performance. However, it is largely unknown how repeatedly performing stressful tasks physically affect police officers in terms of heart rate and pupil diameter properties. Psychological stress is commonly assessed by monitoring the changes in these biomarkers. Heart rate and pupil diameter was measured in 12 male police officers when performing a sequence of four stressful tasks, each lasting between 20 and 130 s. The participants were first placed in a dimly illuminated anteroom before allowed to enter a brightly lit room where a scenario was played out. After each task was performed, the participants returned to the anteroom for about 30 s before performing the next sequential task. Performing a repeated sequence of stressful tasks caused a significant increase in heart rate (*p* = 0.005). The heart rate started to increase already before entering the scenario room and was significantly larger just after starting the task than just before starting the task (*p* < 0.001). This pattern was more marked during the first tasks (*p* < 0.001). Issuance of a verbal “abort” command which terminated the tasks led to a significant increase of heart rate (*p* = 0.002), especially when performing the first tasks (*p* = 0.002). The pupil diameter changed significantly during the repeated tasks during all phases but in a complex pattern where the pupil diameter reached a minimum during task 2 followed by an increase during tasks 3 and 4 (*p* ≤ 0.020). During the initial tasks, the pupil size (*p* = 0.014) increased significantly. The results suggest that being repeatedly exposed to stressful tasks can produce in itself an escalation of psychological stress, this even prior to being exposed to the task. However, the characteristics of both the heart rate and pupil diameter were complex, thus, the findings highlight the importance of studying the effects and dynamics of different stress-generating factors. Monitoring heart rate was found useful to screen for stress responses, and thus, to be a vehicle for indication if and when rotation of deployed personnel is necessary to avoid sustained high stress exposures.

## Introduction

The term “stress” can be used to describe the effects of many different strains on our physiology, e.g., stress due to illnesses, temperature and dehydration (Ulrich-Lai and Herman, 2009). In this article, we will focus on two specific types of stress. The first category is physical stress, which is induced by physical activity. Physical stress affects many of our neurobiological functions, causing e.g., increased heart rate, breathing rate, vasodilation (widening of blood vessels) and increased body temperature (Burton et al., 2004). The second stress category is commonly termed “psychological stress” or as “the stress response” (Bracha, 2004; Qin et al., 2009; LeDoux and Pine, 2016). The stress response is produced by reflexive neural (autonomic sympathetic) and neuroendocrine (hypothalamic-pituitary-adrenal) processes, and is activated by external (e.g., a barking dog) or internal cognitive information (e.g., memories or nightmares), that are subconsciously or consciously perceived as a threat (Ulrich-Lai and Herman, 2009). Hence, a stress response might be activated subconsciously, and thus, the physical and neurological processes activated by stress might be initiated before a human in his/her conscious mind have identified a threat motivating feeling fear or anxiety (LeDoux, 2000; Pariyadath and Eagleman, 2007; LeDoux and Pine, 2016).

A stress response can be activated regardless of current physical activity and affect simultaneously several human functions. Both physical and psychological stress (the stress response) increase heart rate, blood pressure and breathing (Freyschuss et al., 1988; Ulrich-Lai and Herman, 2009). However, the psychological stress response may also cause several physical changes with sometimes opposite effects than those caused by physical stress, e.g., a narrowing of peripheral blood vessels (vasoconstriction) instead of a widening (Ulrich-Lai and Herman, 2009). Psychological stress and the stress response can in different contexts either improve or impair the perceptive, cognitive and motor performance depending on factors like the properties of the perceived threat and the strength of the stress response (Siddle, 1995; Stefanucci et al., 2008; Qin et al., 2009; Bertilsson et al., 2013; Petersson et al., 2017). The performance typically follows an inverted U-function with increasing stress levels, i.e., the performance increases when the stress levels increase from low to moderate and thereafter decreases if the stress levels increase from moderate to high levels (Yerkes and Dodson, 1908; Siddle, 1995; Grossman and Christensen, 2004). Even a subconsciously activated reflexive stress response will also be different in strength depending on the fast subconscious threat level assessment (LeDoux, 2000; LeDoux and Pine, 2016).

An issue that to date has received little attention is that police officers often are exposed to repeated stressful events over shorter (minutes) and longer (hours) periods (Gyamfi, 2012). Police officers often have to solve stressful situations demanding situational awareness, decision-making skills and motor skills (Gächter et al., 2011). Repeated stress exposures has been shown to causes cognitive impairment in rats (Yuen et al., 2012), and similar effects has been detected in humans (Phelps and LeDoux, 2005). Moreover, high levels of stress may cause perceptual and memory distortions (Artwohl, 2002; Qin et al., 2009; Dahl et al., 2018). The long term effects of continuous psychological stress or not reaching homeostasis for longer periods can be dire, with occupational stress burnout (Gächter et al., 2011), e.g., thinning of brain prefrontal and temporal cortical regions and increased amygdala volume (Savic, 2015). Exposures to traumatic events may also increases the susceptibility for posttraumatic stress syndrome (Bomyea et al., 2012). Whether accumulative effects of repeated stressful exposures can be detected using biomarkers heart rate and pupil diameter monitoring is unknown.

To enable police officers to train performing demanding skills when under stress, realistic scenario training might be an important vehicle to inoculate police officers to the often debilitating effects of strong stress responses (Saunders et al., 1996; Bertilsson et al., 2017; Petersson et al., 2017). However, if the training scenarios are too difficult and cause strong feelings of failure or pain, the scenario training may instead produce fear conditioning (Atkins and Norris, 2004), and thus, a worse performance in stressful situations. On the other hand, if the scenarios are too easy and evoke no stress, the participants may not gain the wanted stress inoculation that produces improved performance in stressful situations (Oudejans, 2008). Hence, it is important that training designers and instructors can continuously monitor the stress response of participants performing a scenario using reliable measures to ensure that the training evokes levels of stress that are optimal for stress inoculation.

One frequently used approach to monitoring stress levels is by using biomarkers, such as heart rate (Freyschuss et al., 1988; Carroll et al., 2000; Sawai et al., 2007). However, one drawback with monitoring heart rate is that both physical and psychological stress may cause increased heart rates. Thus, if the training scenario includes performing various kinds of physical activity, the heart rate might be an ambiguous source of information for describing levels of stress. A workaround might be that the participants perform the physical activity alone without active stressors at a separate occasion, thereby allowing a baseline heart rate level caused by performing the physical tasks to be determined (Bertilsson et al., 2013). However, it is doubtful that a good baseline can be established using this method because also without active stressors in a scenario, subjects might feel psychological stress, e.g., from performing tasks in front of experts and colleagues. Another method to assess stress levels is to record the amount of cortisol (Kirschbaum et al., 1995), a stress hormone released through the neuroendocrine (hypothalamic-pituitary-adrenal) system as a part of the psychological stress response (Ulrich-Lai and Herman, 2009). However, monitoring cortisol levels requires access to a laboratory facility. Moreover, it may take more than 20 min until a stress response produce detectable increased levels of cortisol and the response amplitude may differ markedly between subjects and tests performed (Kirschbaum et al., 1995; Bozovic et al., 2013). Hence, cortisol can be used to determine that a stress response has occurred, but provide a poorer measure of when it occurred and what caused the stress response to occur.

Pupil diameter is another commonly used biomarker (Beatty, 1982; Pedrotti et al., 2014) that reflects stress through a sympathetic nerve response that dilates the pupil (Hall and Chilcott, 2018; Wang et al., 2018). A problem with using pupil size as an index of the stress response is that ambient light levels also have a strong effect on pupil diameter through the parasympathetic pupillary light reflex (Bressloff, 1996; Pong and Fuchs, 2000; Hall and Chilcott, 2018). Hence, a short glance toward a lamp, for instance, will induce a large change in the size of the pupil.

The study objective was to determine whether repeatedly performing moderately stressful tasks, executed in a rapid sequence with only a brief rest between tasks, caused an altered stress response as assessed by the biomarkers heart rate and pupil diameter. Another objective was to determine if both biomarkers described the stress response process similarly or reflected different characteristics, i.e., suggesting an individual practical usefulness. Our hypothesis is that both heart rate and pupil diameter will increase when repeatedly performing moderately stressful tasks, but not necessarily in an identical way.

## Materials and Methods

Experiments were performed in accordance with the Helsinki declaration and the recorded data was handled according to the protocol approved by the Ethics Review Board at the Lund University, Sweden (Dnr 2014-36). All participants provided written informed consent.

### Subjects

Twelve healthy male subjects [age *M* = 30.7 (SD 3.2) years] participated in the study, but heart rate recordings from one subject and pupil diameter recordings from another subject had to be excluded due to recording malfunctions. All participants were experienced police officers with at least 5 years prior experience of field duty work. Only fully healthy subjects were allowed to take part in the test scenarios. All subjects had normal visual acuity.

### Equipment

**FIGURE 1:**
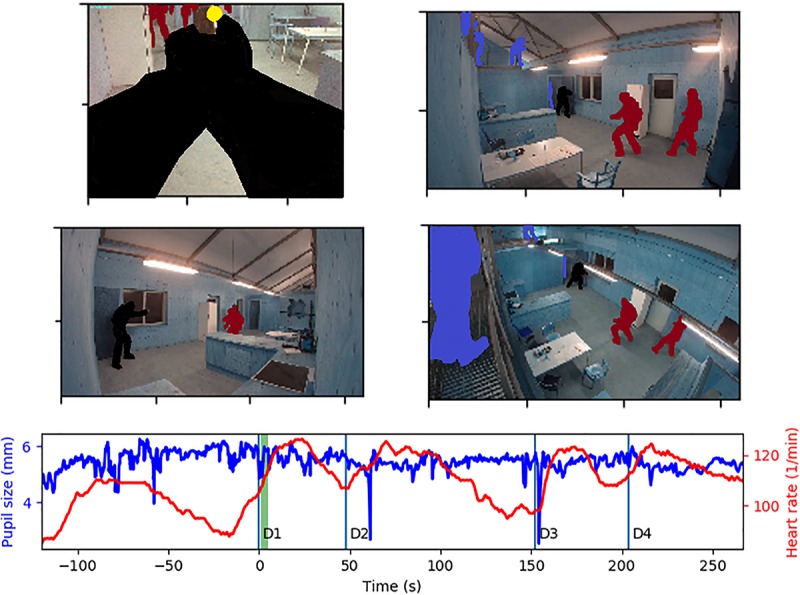
The participant’s performance of the tasks was monitored by a forward-facing camera mounted on the SMI glasses, and three go-pro cameras mounted in the room. The study participant is marked in black, his gaze location is a yellow dot (top-left figure), the scenario figurants are marked in dark-red and the scenario instructors are marked in blue. The participant’s pupil diameter was monitored by the SMI glasses and a Bioharness equipment recorded heart rate. The period marked in green in the diagram shows from what period the images displayed originate. The onsets of task 1–4 are denoted as D1–D4 in the diagram.

Heart rate, breathing rate and body posture were recorded with a Zephyr Bioharness^®^ 2.0 (Zephyr technology corporation, Annapolis, MD, United States), and pupil diameter was recorded with eye tracking glasses from SensoMotoric Instruments (SMI), Berlin, Germany. Additionally, the subjects’ performance in the scenario room was monitored by three Go-Pro 3.0 cameras that which also recorded sound. The Go-Pro cameras were placed in different positions so that the actions of all actors in the scenario was always recorded by at least one Go-Pro camera, see Figure 1. Before the task sequence started, a three-point calibration of the SMI glasses was performed followed by manual inspection of the calibration accuracy by asking the participant to fixate another set of points on a wall. Additionally, a specific movement task was performed to enable time-synchronization of the heart- and breathing rate recordings made with the bioharness and the pupil diameter recordings provided by the eye-tracking glasses. The bioharness system sampled the ECG activity at 250 Hz and the SMI glasses recorded the pupil diameter at 30 Hz.

### Procedure

Four different tasks were performed by the participants in a fixed sequence (see Table 1). Before starting the first task scenario, the participants were sitting down resting for at least 10 min listening to music. The first scenario started shortly after the participants were outfitted with the recording equipment (see “Equipment” below), and the equipment had been calibrated and synchronized together. The participants were also equipped with a SigSauer^®^ pistol adapted for Simunition^®^ cartridges and a pepper spray canister that for training purposes contained water without Oleoresin Capsicum (OC). Each task was preceded by the participant standing for about 30 s in a dimly lit (4.6 Lux) anteroom. All tasks started when participants opened a door and quickly entered the scenario room, where a specific scenario started to play out immediately including 2 or 3 human figurants. When the door to the brighter lit scenario room was opened, the illumination the participant was exposed to first increased to 191 Lux. After completely having entered into the scenario room, in which the scenario played out, the participant was exposed to about 385 Lux illumination. When a scenario instructor judged the policing task to be completed, he terminated the task with a verbal “abort” command, and the participant returned to the anteroom for a brief rest lasting about 33 s (*M* = 33.2, SD = 9.4 s) before the next task commenced. The subject received no detailed instructions about the scenario to address, and was merely asked to deal with the situation.

**TABLE 1:** Scenario description, duration, and rest in between performing the tasks.

**Task**	**Scenario description**	**Duration [Mean (SD)] (seconds)**	**Time interval to next scenario [Mean (SD)] (seconds)**
1.	**Immediate** threat encounter scenario; Hostage situation where a person holds a knife against another person’s throat from behind. When the police officer enters the room, the person with the knife let go of the hostage and walks toward the police officer.	26 (8)	32 (11)
2.	**Delayed** threat encounter scenario; A person is sitting at a table and acts calmly when the police officer enters the room. Suddenly an angry non-compliant person appears from behind a fridge with a knife and moves slowly toward the police officer.	34 (22)	34 (12)
3.	**Immediate** threat encounter scenario; Hostage situation with a gun threat. A person is sitting in a chair and another person holds him from behind and points a gun point blank at his head and then turns the gun against the police officer.	25 (6)	33 (4)
4.	**Delayed** threat encounter scenario; Compliant persons in a room with two dangerous objects hidden in plain sight. When the police officer opens the door, one person is standing to his right and suddenly another curious person appears from behind the opened door.	136 (48)	

The physical activity during and between tasks was monitored by recording the number of steps made during each time window analyzed. The steps made was determined from inspecting the recordings from the four cameras used in the test setup. The policing actions performed by the participant during all scenarios involved at most medium walking cadence (i.e., ≤1.3 steps/s) (see Table 2). The participants continued to carry the bioharness equipment, monitoring the heart rate, for about 5 h after the four scenario tasks were completed. The lowest heart rate recorded during this 5-h period was denoted the heart rate at rest (see Table 3).

**TABLE 2:** Physical activity.

**Walking cadence ^a,b^**		**Pre Onset**	**Post Onset**	**Scenario activity^c^**	**Pre Offset**	**Post Offset**	**Between scenario^d^**
Task 1	Steps/s^e^	0.0 (0.0)	0.7 (0.1)	0.4 (0.1)	0.3 (0.1)	1.0 (0.1)	0.0 (0.0)
	Duration	5.0 (0.0)	5.0 (0.0)	15.3 (2.2)	5.0 (0.0)	5.0 (0.0)	32.1 (3.1)
Task 2	Steps/s^e^	0.0 (0.0)	0.9 (0.1)	0.4 (0.1)	0.7 (0.1)	1.2 (0.1)	0.0 (0.0)
	Duration	5.0 (0.0)	5.0 (0.0)	22.9 (6.0)	5.0 (0.0)	5.0 (0.0)	34.8 (3.2)
Task 3	Steps/s^e^	0.0 (0.0)	0.6 (0.1)	0.6 (0.1)	0.7 (0.1)	1.1 (0.1)	0.1 (0.0)
	Duration	5.0 (0.0)	5.0 (0.0)	15.2 (1.6)	5.0 (0.0)	5.0 (0.0)	31.9 (1.3)
Task 4	Steps/s^e^	0.0 (0.0)	0.4 (0.1)	0.4 (0.1)	0.7 (0.1)	1.3 (0.1)	–
	Duration	5.0 (0.0)	5.0 (0.0)	118.8 (15.0)	5.0 (0.0)	5.0 (0.0)	–

*^*a*^Physical activity described as walking cadence steps/second, mean (SEM). ^*b*^Duration of each analyzed time windows, mean (SEM). ^*c*^Scenario activity = Activity between Post Onset and Pre Offset. ^*d*^Between scenario = Activity between Post Offset and the next task Pre Onset. ^*e*^For comparison, the medium walking cadence for a healthy person is about 1.3–1.6 steps/s Tudor-Locke et al. (2018).*

**TABLE 3:** Physical characteristics.

**Physical characteristics ^a,b^**	**Age**	**HR Rest**	**HR Pre Onset Task 1**	**HR Task 1^c^**	**HR Task 2^c^**	**HR Task 3^c^**	**HR Task 4^c^**
	30.7 (1.1)	71.3 (4.4)	105.9 (3.8)	119.2 (5.3)	119.6 (4.3)	122.1 (4.1)	117.5 (3.7)

*^*a*^Physical characteristics are described as mean (SEM). ^*b*^The duration of the Pre Onset window is 5 s. ^*c*^The duration of tasks 1–4 is the active scenario time from Onset to Offset, see Table 2 for details.*

### Analysis

**FIGURE 2:**
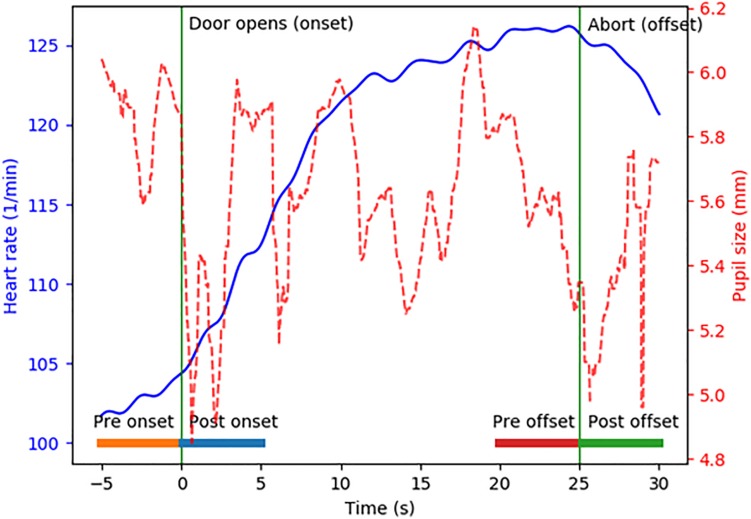
Example of a participant’s heart rate (blue) and pupil diameter (red) when performing a task. The 5-s analyses windows submitted to detailed analyses are marked by the horizontal bars denoted Pre onset, Post onset, Pre offset, and Post offset.

The heart rate and pupil diameter were analyzed offline by a custom-made program. The Onset time of each task was defined as when the door between the anteroom and scenario room was opened, and thus, when the scenario was revealed to the participant. The Offset time of the task was defined as the moment when the scenario instructor terminated the task with a verbal “abort” command. For both heart rate and pupil diameter, analyses windows were defined as a 5-s period before the task onset (denoted Pre Onset) and as a 5-s period after the task onset (Post Onset). Additionally, analyses windows were defined as a 5-s period before the task offset (denoted Pre Offset) and as a 5-s period after the task offset (Post Offset). These analysis windows are illustrated in Figure 2.

### Statistical Analysis

The heart rate and pupil diameter during the four repeated tasks were analyzed using repeated measures GLM ANOVA. The main factors and factor interactions analyzed were: “Repetition” (Task 1–4; d.f. 3); and “Window” which was evaluated for three different pairs of analysis windows [door opening (Pre Onset vs. Post Onset), task execution (Post Onset vs. Pre Offset) and abort command (Pre Offset vs. Post Offset); d.f. 1]. The repeated measures GLM ANOVA analysis method was used after ensuring that all dataset combinations analyzed in the study with this statistical method produced model residuals that had normal or close to normal distribution (Altman, 1991).

Wilcoxon matched-pairs signed-rank tests (Exact sig. 2-tailed) were used for within-group *post hoc* comparisons, i.e., analyzing the accumulated changes from task 1 to task 4. Moreover, as part of the *post hoc* evaluation, the best fitting dynamic patterns and time constants describing the changes in heart rate and pupil diameter during the four time windows (Pre Onset, Post Onset, Pre Offset, and Post Offset) during each task were determined by using regression models after evaluating different regression models for best fit (linear, exponential etc.). A linear regression model was found to describe best the changes in heart rate whereas an exponential regression model was found to describe best the changes in pupil diameter over time.

Spearman’s two-tailed correlation analyses were used for determining relationships between the subject’s age and heart rate at rest and heart rate across performing the repeated tasks. Moreover, correlations analysis was also used to determine relationships between the heart rate recorded across performing the repeated tasks.

In all analyses, *p*-values <0.05 or *p* < 0.01 were considered significant after Bonferroni correction, depending on the number of within-subject tests performed. The Shapiro-Wilk test revealed that some datasets were not normally distributed and that normal distribution could not be obtained by log-transformation. Thus, non-parametric statistical methods able to appropriately handle non-normal distributions were used in all *post hoc* evaluations (Altman, 1991).

A sample size analyses, using the statistical package G-power^TM^ , were performed on the heart rate recorded during rest and average heart rate while performing each of the four tasks. The analysis revealed an effect size for each of the tasks of 2.6 (task 1); 2.9 (task 2); 3.3 (task 3), and 2.9 (task 4) which shows that with the *p*-value set to 0.05 (2-tailed), our study would require *n* = 4 subjects to reach a power value of 0.8 for this parameter.

The statistical analyses were performed with SPSS version 24 and the power analysis was performed with GPower version 3.1.9.4.

## Results

### Test Condition Evaluation

The resting time between performing any of the four stressful tasks was not significantly different (*p* ≥ 0.400), see Table 1. Moreover, the duration of the three first tasks was not significantly different (*p* ≥ 0.158). However, the duration of the fourth and last task was significantly longer than that of all the three initial tasks (*p* < 0.001).

### Physical Activity During Each of the Time Windows Investigated

The test subjects were always standing still during the 5 s Pre Onset window 0.0 (0.0) steps/s, see Table 2. The physical activity across all scenarios and scenario windows Post Onset, Scenario activity and Pre Offset, were on average not exceeding 0.9 (0.1) steps/s. The Post Onset physical activity was significantly larger during task 2 compared with task 4 (*p* = 0.005). The Post Offset activity consisted mostly of returning back to the anteroom, which at most reach an activity of 1.3 (0.1) steps/s. During the between scenario times, the test subjects were typically standing still 0.1 (0.0) steps/s. For comparison, the medium walking cadence for a healthy person is about 1.3–1.6 steps/s (Tudor-Locke et al., 2018).

The heart rate recorded during rest was significantly lower than during Pre Onset of task 1 (*p* < 0.001) and significantly lower than during any of the tasks 1–4 (*p* < 0.001), see Table 3. Moreover, the heart rate recorded during Pre Onset of task 1 (*p* < 0.001) was significantly lower than during any of the tasks 1–4 (*p* < 0.010). The average heart rate recorded during tasks 1–4 were not significantly different between each other.

Correlation analyses between the subject’s age and heart rate during each of the windows analyzed revealed no significant relationship. However, the heart rate at rest was significantly correlated to the heart rate at Pre Onset of task 1 (*p* = 0.014, *R* = 0.711). Moreover, the heart rate at Pre Onset of task 1 were significantly correlated to the average heart rates during tasks 1–3 (*p* ≤ 0.013, *R* ≥ 0.718). Finally, the average heart rates recorded during tasks 1–4 were significantly correlated to each other (*p* ≤ 0.011, *R* ≥ 0.727).

### Effects of Repetition and Window on Heart Rate When Repeatedly Performing Tasks

**FIGURE 3:**
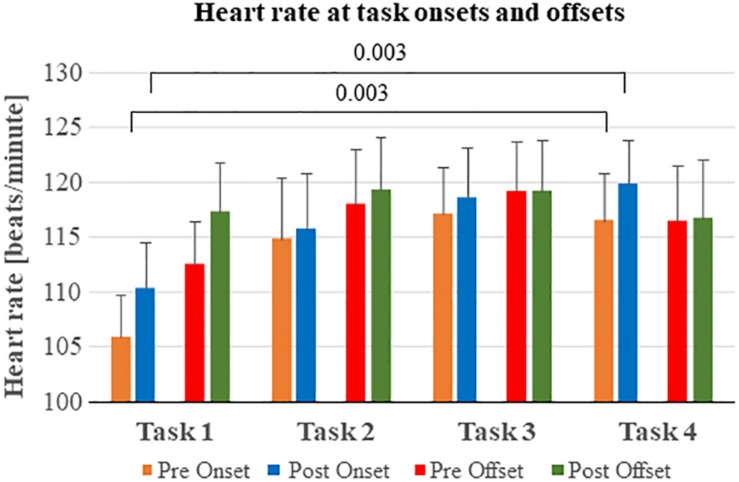
Heart rate before and after task onsets and offsets. The heart rate became increasingly larger at task onset by each test performed. The heart rate continued to increase until just before task offset, but primarily while performing the first tasks in the series.

Repeatedly performing stressful tasks significantly increased the heart rate during door opening across the four tasks (*p* = 0.005) (Figure 3 and Table 4). Moreover, the heart rate was significantly larger post onset than pre onset for all tasks (*p* < 0.001). The interaction between main factors Repetition x Window revealed that the heart rate pre onset was lower than post onset at the initial tasks but the differences decreased during the last tasks (*p* < 0.001).

**TABLE 4:** Effects on the heart rate of performing repeated tasks.

**Task phase**	**Repetition^a^**	**Window^a^**	**Repetition × Window^a^**
Door opening (pre onset vs. post onset)	**0.005 [12.7]**	**<0.001 [21.1]**	**<0.001 [22.3]**
Task execution (post onset vs. pre offset)	0.068 [4.2]	0.766 [0.1]	0.345 [1.0]
Abort command (pre offset vs. post offset)	0.085 [3.7]	**0.002 [18.0]**	**0.002 [17.0]**

*^*a*^*p*-values and(*F*-values). The notation “<0.001” means that the *p*-value is smaller than 0.001. Bold values indicate significance.*

The heart rate values recorded at scenario pre offsets were significantly lower than the heart rate recorded after receiving the command “abort” (*p* = 0.002). However, the interaction between main factors Repetition x Window revealed that this difference decreased significantly over repeated tasks (*p* = 0.002).

*Post hoc* analyses were performed to determine the total accumulated effects of performing repeated tasks (Figure 3). The heart rate at pre onset was significantly lower at task 1 compared with task 4 (*p* = 0.003). Moreover, the heart rate at post onset was significantly lower at task 1 compared with task 4 (*p* = 0.003).

*Post hoc* regression analysis of the heart-rate time series during the 5-s windows revealed for task 1 significant heart rate increases during the pre onset (rate of change 43.6 beats per minute (bpm), *p* < 0.001) and the post onset windows (64.0 bpm, *p* < 0.001) (Table 5). A similar significant heart rate increase was found during task 4 pre onset (33.1 bpm, *p* = 0.017) and post onset (41.8 bpm, *p* = 0.001) windows. Moreover, a significant heart rate increase during pre offset (52.3 bpm, *p* < 0.001) and post offset (54.2 bpm, *p* < 0.001) windows was found during task 1.

**TABLE 5:** Linear regression analysis of temporal trends in heart rate for each task phase.

	**Window**	***p*-value^a^**	**Constant^b^**	**Time constant^c^**
Task 1	Pre Onset	**<0.001 [12.6]**	**104.1**	**43.6**
	Post Onset	**<0.001 [22.1]**	**107.7**	**64.0**
	Pre Offset	**<0.001 [16.5]**	**110.4**	**52.3**
	Post Offset	**<0.001 [13.6]**	**115.1**	**54.2**
Task 2	Pre Onset	0.521 [0.4]	114.4	11.3
	Post Onset	0.257 [1.3]	115.0	18.4
	Pre Offset	0.250 [1.3]	117.3	18.5
	Post Offset	0.607 [0.3]	118.9	8.1
Task 3	Pre Onset	0.402 [0.7]	116.6	11.7
	Post Onset	0.087 [2.9]	117.6	25.4
	Pre Offset	0.585 [0.3]	118.8	8.0
	Post Offset	0.134 [2.3]	119.6	23.3
Task 4	Pre Onset	**0.017 [5.7]**	**115.1**	**33.1**
	Post Onset	**0.001 [10.6]**	**118.1**	**41.8**
	Pre Offset	0.678 [0.2]	116.2	6.7
	Post Offset	0.889 [0.0]	116.9	−2.4

*^*a*^*p*-values and (*F*-values). The notation “<0.001” means that the *p*-value is smaller than 0.001. ^*b*^Heart rate starting value for window. ^*c*^Linear change in heart rate (beats per minute). Bold values indicate significance.*

### Effects of Repetition and Window on Pupil Diameter When Repeatedly Performing Tasks

**FIGURE 4:**
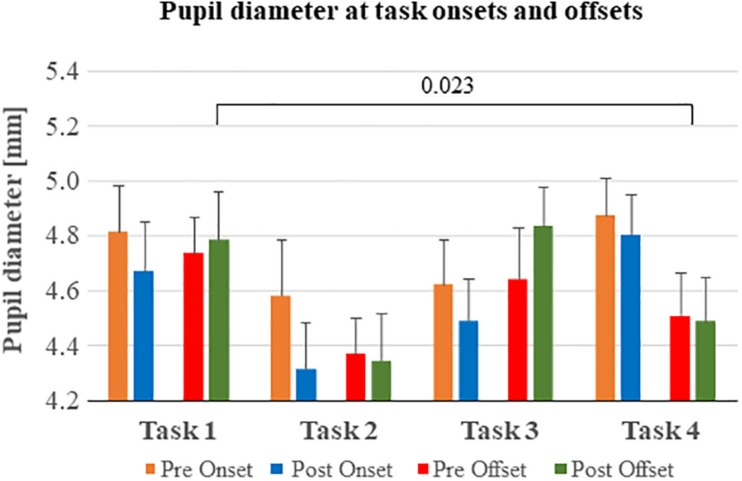
Pupil diameter before and after task onsets and offsets. The pupil diameter reached a minimum in all analysis windows (Pre onset, Post onset etc.) during task 2. In tasks 1 and 3, the participant encountered an immediate threat, in most cases within the first 5 s of scenario. During tasks 2 and 4 the threat appeared delayed more than 5 s into the scenario.

Repeatedly performing tasks significantly changed the pupil diameter at the door opening (pre onset and post onset) in a complex pattern, i.e., the pupil diameter reached a minimum during task 2 and increased diameter again during task 3 and 4 (*p* = 0.008) (Figure 4 and Table 6).

**TABLE 6:** Effects on the pupil diameter of performing repeated tasks.

**Task phase**	**Repetition^a^**	**Window^a^**	**Repetition × Window^a^**
Door opening (pre onset vs. post onset)	**0.008 [15.1]**	0.160 [2.6]	0.653 [0.2]
Task execution (post onset vs. pre offset)	**0.020 [8.4]**	0.583 [0.3]	**0.014 [9.7]**
Abort command (pre offset vs. post offset)	**<0.001 [35.3]**	0.402 [0.8]	0.176 [2.1]

*^*a*^*p*-values and (*F*-values). The notation “<0.001” means that the *p*-value is smaller than 0.001. Bold values indicate significance.*

During task execution (post onset and pre offset), the pupil diameter changed in the same complex pattern of reaching the smallest pupil diameter while performing task 2 followed by an increase during tasks 3 and 4 (*p* = 0.020). However, the interaction between main factors Repetition × Window suggests that the pupil diameter at post onset was smaller than pre offset during the first tasks but changed during the last task 4 (*p* = 0.014).

Finally, during the abort command (pre offset and post offset) the pupil diameter changed in the same complex pattern as found during the other task phases in that it reached the smallest pupil diameter while performing task 2 followed by an increase during tasks 3 and 4 (*p* < 0.001).

*Post hoc* analyses were performed to determine the total accumulated effects of performing repeated tasks, in terms of comparing the pupil diameter at the first and last of the four repeated tasks (Figure 4). The pupil diameter at post offset was significantly reduced between task 1 and test 4 (*p* = 0.023).

*Post hoc* regression analyses of pupil diameter development revealed a significant decrease in pupil diameter during pre onset before task 1 (rate of change = −0.018, *p* < 0.001), before task 2 (−0.013, *p* < 0.001) and before task 4 (−0.012, *p* < 0.001), whereas the pupil diameter increased significant pre onset before task 3 (0.009, *p* < 0.001) (Table 7). However, a significant pupil diameter increase was detected during post onset of task 1 (rate of change = 0.015, *p* < 0.001), task 2 (0.005, *p* = 0.039) and task 3 (0.009, *p* = 0.002), whereas a pupil diameter decrease was detected during post onset of task 4 (−0.007, *p* < 0.001).

**TABLE 7:** Exponential regression analysis of temporal trends in pupil diameter for each task phase.

	**Window**	***p*-value^a^**	**Constant^b^**	**Time constant^c^**
Task 1	Pre Onset	**<0.001 [57.8]**	**5.02**	**−0.018**
	Post Onset	**<0.001 [39.2]**	**4.54**	**0.015**
	Pre Offset	0.912 [0.0]	4.74	0.000
	Post Offset	**0.018 [5.6]**	**4.76**	**0.005**
Task 2	Pre Onset	**<0.001 [18.3]**	**4.67**	**−0.013**
	Post Onset	**0.039 [4.3]**	**4.30**	**0.005**
	Pre Offset	**<0.0001 [16.4]**	**4.49**	**−0.009**
	Post Offset	0.913 [0.0]	4.38	0.000
Task 3	Pre Onset	**<0.001 [12.1]**	**4.53**	**0.009**
	Post Onset	**0.002 [9.9]**	**4.44**	**0.009**
	Pre Offset	0.150 [2.1]	4.66	0.003
	Post Offset	**0.029 [4.8]**	**4.83**	**0.004**
Task 4	Pre Onset	**<0.001 [39.3]**	**5.02**	**−0.012**
	Post Onset	**<0.001 [12.3]**	**4.90**	**−0.007**
	Pre Offset	0.780 [0.1]	4.52	**−**0.001
	Post Offset	**0.003 [8.8]**	**4.54**	**−0.006**

*^*a*^*p*-values and (*F*-values). The notation “<0.001” means that the *p*-value is smaller than 0.001. ^*b*^Pupil diameter starting value for window. ^*c*^Exponential change in pupil diameter per second. Bold values indicate significance.*

The pupil diameter changes were disparate during task offsets. A significant pupil diameter decrease was found during pre offset of task 2 (rate of change = −0.009, *p* < 0.001). However, a significant pupil diameter increase was detected during post offset of task 1 (rate of change = 0.005, *p* = 0.018) and task 3 (0.004, *p* = 0.029), and a pupil diameter decrease was detected during post offset of task 4 (−0.006, *p* = 0.003).

## Discussion

Police officers are often exposed to stressful situations in their daily work. Little is, however, known about how repeated exposure to stressful situations that require police intervention influence physiological measures of stress. The heart rate and pupil size were measured in twelve experienced police officers while they performed policing tasks during four short scenarios containing stressful problems and threats to solve.

### Effects of Repeatedly Performing Stressful Tasks on Heart Rate

Our results show that the heart rate of police officers gradually increased at the onset of the tasks across the four tasks, see Figure 3. Moreover, the heart rate was significantly higher just after the task onset compared to just before the task onset. However, the differences in heart rate before and after task onset decreased across the repetitions due to an increase in pre-task heart rate. Hence, the heart rate recordings suggest that the stress levels escalate when repeatedly performing stressful tasks, manifested mainly as a higher heart rate at the onset of the tasks. This said, after the initial escalation in heart rate during the first tasks, the heart rate seems to approach a new higher steady state level at task onset and offset already while performing task 3 and task 4.

That the first test scenario induced the largest increase in heart rate is similar to the findings by Kirschbaum et al. (1995), who reported an increased concentration of cortisol due to stress induced by speaking and solving numerical problems in front of an audience. However, regression analyses investigating changes in heart rate during the analyses windows suggest that during two of the tasks (task 1 and 4), the increase in heart rate started already before the task onset (Pre Onset) and continued to increase at an even faster rate after the door had opened (Table 5). This increase was observed while the participants were standing completely still (Pre Onset), or took a few steps into the scenario room at an average walking cadence not exceeding 0.9 steps/second (Post Onset) (see Table 2). For comparison, the moderate walking cadence for a healthy person is about 1.3–1.6 steps/s (Tudor-Locke et al., 2018). Moreover, the participants had been resting for at least 10 min, sitting down, prior to performing the four tasks. Hence, since all participants in the study had an above average physical strength and cardiovascular endurance level, it is unlikely that the low physical activity during the tasks would lead to heart rates of about 120 bpm in tasks 3 and 4, but instead likely reflect an induced stress response. Moreover, the average 106 bmp heart rate recorded during Pre Onset of task 1 compared with the average heart rate at rest of 71 bpm suggest that the participants were stressed already before performing any tasks. Nevertheless, that the average heart rate increase to 120 bmp during the sequence suggests that the participants experienced not more than a moderate threat from performing the tasks.

An unexpected finding was the close relationship between the heart rate at rest, just prior to performing the first task and heart rate across the tasks performed. These results suggest that the level of heart rate include a substantial individual component that persist also when affected by stress. Moreover, these findings also suggest that the changes recorded in heart rate before and while performing the tasks were not random but included a significant systematic behavior.

There were no significant changes in heart rate over the course of the tasks (Post onset vs. Pre Offset). However, the “abort” command, which terminated a scenario, was associated with an overall increase in heart rate across the repetitions. This heart rate response was more marked during the initial tasks but declined when reaching the last tasks 3 and 4. This response, predominantly found during the first tasks, might be a startle effect from that the instructor intervening in the scenario. When performing the last tasks, the participants knew that the scenarios would end this way, and the intervention therefore did not elicit a similar response.

### Effects of Repeatedly Performing Stressful Tasks on Pupil Size

The interpretation of pupil activity was in general more complex compared to heart rate. During task onsets, there was a significant difference in pupil size across the task sequence. However, unlike heart rate, *post hoc* tests did not show a gradual increase in pupil size over tasks, but rather unsystematic variations from task to task, where the smallest pupil sizes were recorded during tasks 2 and 4. Similar patterns were found for the other task phases (Task execution, Abort command). It is difficult to explain why we see these differences in pupil size across tasks and task phases, and they may simply be related to the natural fluctuations in pupil size, which can reach up to 0.5 mm, in combination with changes in pupil size due to scenario-dependent differences in gaze direction (Centeno et al., 2011). However, the pupil diameter tended to respond differently to differences in the scenario content, e.g., the pupil diameter inclined to be larger when the threat more often appeared immediately, as in scenario 1 and 3 and smaller when the threat appeared delayed, as in scenario 2 and 4. Hence, the differences in scenario content suggests that the biomarkers heart rate and pupil diameter respond differently to different features in the tasks to perform under various levels of stress.

Moreover, the pupil diameter did not change to the extent expected in response to the changes in illumination. The average pupil diameter across all tasks was about 4.7 mm (SD 0.8 mm) at 4 lux, 4.6 mm (SD 0.5 mm) at 191 lux and 4.6 mm (SD 0.5 mm) at 385 lux. For reference, when investigating the effects of different illumination levels on pupil diameter under non-stressful conditions, Gholami et al. (2018), found that the average pupil diameter was about 6.4 mm (SD 1.0 mm) at 4 lux, 5.7 mm (SD 0.9 mm) at 40 lux and 4.3 mm (SD 0.8 mm) at 400 lux. When entering the scenario room (Post Onset), one would expect the pupils to constrict due the much larger illumination in the scenario room (385 lux) compared to the dimly lit anteroom (4.6 lux). Interestingly, however, during the first three tasks, the regression analyses revealed that the pupils dilated significantly during the post onset analysis window, which is contrary to what would be expected due to the sharp increase in illumination. A possible explanation to our findings could be that the parasympathetic pupillary light reflex was overpowered by a stronger sympathetic nerve response, causing the pupils to dilate in spite of the participants entering a room with much higher illumination (Beatty, 1982; Pedrotti et al., 2014).

#### Potential Role of Homeostasis

The stress response has a fast autonomic sympathetic part and a somewhat slower endocrine or hormonal part. The sympathetic response uses neural pathways to activate basic physiological functions such as increased heart rate, pupil dilation, blood pressure, breathing rate and vasoconstriction (Purves et al., 2018). A sympathetic pathway to the adrenal medulla starts the release of adrenaline and noradrenaline to the blood system, which will strengthen and prolong the stress response effects. However, it takes several seconds to increase the stress hormone blood concentrations. Yet another stress system is the hypothalamic-pituitary-adrenal cortex or HPA-axis that through release of hormones in the blood system activates the adrenal cortex to among others release the stress hormone cortisol in to the blood system, which increases the ability to maintain a stress response over longer time if necessary. If the stress response is not inhibited due to obvious threat removal within about 15–30 s the stress hormone levels in the blood system will have reached levels to a point that even if the threat disappears the effects will last up to an hour or more before homeostasis is reached. High levels of cortisol can take hours to reduce (Hallett, 2012; Bozovic et al., 2013; Osborne et al., 2015; Wilkinson and Imran, 2019). Thus, even if the first three tasks were performed within about 30 s, this duration might be too long to enable a reset of the stress response systems. Concomitantly, the anticipation itself of coming unknown problems and uncertainty about how their performance will be judged may also contribute and explain the apparent increase of heart rate after the given abort command. Moreover, the only about 30 s of rest between tasks would not suffice for a recovery from a stress response that progressed into its long-term stress hormone phase. Hence, the study findings of an accumulated heart rate increase from repeatedly performing stressful tasks are in line with a hypothesis of an incomplete homeostasis between tasks performed. However, more research is needed to determine the role of homeostasis processes during different stress levels and threat durations, and about whether extensive high stress training may influence the neurobiological properties of the stress response and the processes of homeostasis.

### Practical Implications

Temporary increased strength and faster sensory information processing during threatening stressful events, can easily be recognized as beneficial from an evolutionary point of view. A similar stress response system can be found in most mammals (Nesse et al., 2016). Psychological stress is a reflexive response caused by threats affecting performance both positively and negatively. Moderate stress levels can heighten awareness and improve performance. Higher stress levels start to impair decision making, decrease shooting performance and is an important cause of friendly fire (Meyerhoff et al., 2004; Oudejans, 2008; Vonk, 2008; Kassam et al., 2009). It should be noted that the scenarios in this study evoked heart rates up to about 120 bmp, suggesting that only a moderate escalated stress response was induced by the used scenarios. However, already at these stress levels starts fine motor skills using smaller muscle groups to deteriorate, which may have implications on the ability to handle, e.g., safety mechanisms requiring precise finger and hand performance (Siddle, 1995). The more severe motor skill effects typically starts to appear at heart rates higher than 145 bpm (Siddle, 1995; Nieuwenhuys et al., 2009), affecting also the dilation of the pupils (Hollingsworth et al., 2009).

Our results show that being repeatedly exposed to stressful tasks can produce an escalation of psychological stress, this even prior to being exposed to the task to perform. In this study the scenarios evoked only moderate stress responses. However, the importance and effects of stress escalation while performing sequential tasks might be of much more marked role in real-life situations where police officers may encounter true risks of being injured or killed. During such circumstance, a further deterioration of perceptual, cognitive and motor performance caused by a stress accumulation might render police forces unable to complete their tasks and unable to handle equipment requiring fine and complex motor skills (Siddle, 1995). Hence, the findings highlight the importance of avoiding sustained exposure to high stress by allowing de-escalating rests between performing stressful tasks, even though this may be difficult to implement in everyday police work (Anderson et al., 2002). In protracted intense situations, e.g., riots or hostage situations, recording heart rate may here be a useful method to monitor for strong stress responses. Moreover, since police training and evaluation often include sequential tasks, the study results may have practical implications when developing police training, planning at strategical as well as operational or tactical levels. Here, it is important to use a suitable biomarker when determining the effectiveness of different scenarios, training programs, and tests, to induce the required levels of stress response to reach specific goals (Murray, 2004; Bertilsson et al., 2013). The knowledge can also be useful in after-action reviews of stressful police interventions.

In this study, the heart rate was found to be the most useful biomarker for monitoring stress levels. The heart rate was comparatively easy to measure with modern wireless equipment and the findings easy to interpret also by novice users. Thus, recording heart rate might be of marked practical value also during real-life incident and police training. Pupil size was also easy to measure in active scenarios, but was somewhat more difficult to interpret. This makes recording pupil size presently more useful in specifically designed experimental research settings. Hence, by developing custom-made equipment and software, objective physical biomarkers might in the future be of marked help as a vehicle to evaluate performance and operative tactics.

### Limitations

The study was performed on a limited number of subjects (*n* = 12), which gives limited opportunities to explore the effects of, e.g., randomized scenario orders. Given the large heart rate increases compared with during rest, the power analyses performed suggest that the number of subjects was enough for performing statistical analyses. Moreover, that the scenarios were different in key aspects, e.g., in that a threat appeared immediately in scenario 1 and 3 and delayed in 2 and 4, had no noticeable effect on heart rate. The accumulated increase in heart rate continued in spite of differences between scenarios, see Figure 3. However, the pupil diameter tended to respond differently to differences in the scenario content, though a systematic response pattern across repeated task could still be determined, see Figure 4. Hence, the fixed scenario test order used in this study helped to reveal that the biomarkers heart rate and pupil diameter may respond differently to different features in an investigation design. That said, more research should be done on larger materials, e.g., using randomized test orders, to explore whether biomarkers systematically respond differently to specific scenario events or assignments.

Another limitation was that human figurants were used in the scenarios. The main reason for this choice was that human figurants would be able to interact with the test subject during the scenario, and thus, produce a more realistic context. However, this also means that although each scenario included the same task to solve, the events did not play out identically. Hence, detailed analysis of heart rate variability, response latencies and detection of startle reactions were difficult within the scope of this study.

Finally, the pre onset heart rate values just before performing task 1 were already then significantly larger than the heart rate at rest. This may have had implications on our results in that the heart rate effects of repeatedly performing stressful tasks if compared with the heart rate levels at Pre Onset of task 1. Hence, that the subjects were physically inactive for at least 10 min prior to performing the tasks did not make the heart rate levels approach the ones recorded at rest.

## Conclusion

When participants performed a sequence of four stressful tasks and were only allowed a brief rest between tasks caused this a significant accumulated increase in heart rate. The pupil diameter also changed significantly but in a complex pattern likely more related to the contexts in the individual scenarios, e.g., whether the threat appeared immediately or was delayed in the scenario. Thus, being repeatedly exposed to stressful tasks can produce in itself an escalation of psychological stress, which highlight the importance of avoiding sustained exposures to high stress by allowing de-escalating rests between stressful tasks. In this study, heart rate was found to be the most useful biomarker for monitoring stress levels. The heart rate was found comparatively easy to measure with modern wireless equipment and the findings easy to interpret also by novice users. Thus, recording heart rate might be of marked practical value also during real-life incident and during police training. Pupil size was also easy to measure in active scenarios, but was somewhat more difficult to interpret. This makes recording pupil size presently more useful in specifically designed experimental research settings. Hence, by developing custom-made equipment and software, objective physical biomarkers might in the future be of marked help when evaluating performance and operative tactics.

## Data Availability

The datasets generated for this study are available on request to the corresponding author.

## Ethics Statement

Experiments were performed in accordance with the Helsinki declaration and the recorded data was handled according to the protocol approved by the Ethics Review Board at the Lund University, Sweden (Dnr 2014-36). All participants provided written informed consent.

## Author Contributions

JB, PJF, MN, and P-AF collected the data aided by MJM and OF. P-AF, MN, and DN carried out the statistical analyses and worked on the draft of the manuscript together with JB. JB performed the first literature search. JB, P-AF, MN, and DN worked on the several drafts of the manuscript. MD, SG, PJF, MM, OF, and MJM revised the manuscript and contributed with literature.

## Conflict of Interest Statement

The authors declare that the research was conducted in the absence of any commercial or financial relationships that could be construed as a potential conflict of interest.
